# The Association Between Vulnerable/Grandiose Narcissism and Emotion Regulation

**DOI:** 10.3389/fpsyg.2020.519330

**Published:** 2020-10-15

**Authors:** Leonie A. K. Loeffler, Anna K. Huebben, Sina Radke, Ute Habel, Birgit Derntl

**Affiliations:** ^1^Department of Psychiatry, Psychotherapy and Psychosomatics, Faculty of Medicine, RWTH Aachen, Aachen, Germany; ^2^JARA-Institute Brain Structure Function Relationship, Research Center Jülich, RWTH Aachen, Aachen, Germany; ^3^Institute of Neuroscience and Medicine 10, Research Center Jülich, Jülich, Germany; ^4^Department of Psychiatry and Psychotherapy, Medical School, University of Tübingen, Tübingen, Germany; ^5^Werner Reichardt Center for Integrative Neuroscience, University of Tübingen, Tübingen, Germany; ^6^LEAD Graduate School and Research Network, University of Tübingen, Tübingen, Germany

**Keywords:** vulnerable narcissism, grandiose narcissism, emotion regulation, reappraisal, suppression, depression, anhedonia, sex

## Abstract

Narcissism has been widely discussed in the context of career success and leadership. Besides several adaptive traits, narcissism has been characterized by difficulties in emotion regulation. However, despite its essential role in mental health, there is little research on emotion regulation processes in narcissism. Specifically, the investigation of not only the habitual use of specific regulation strategies but also the actual ability to regulate is needed due to diverging implications for treatment approaches. Thereby it is important to differentiate between vulnerable and grandiose narcissism as these two phenotypes might be related differently to regulation deficits. The aim of this study was to examine the association between grandiose and vulnerable narcissism and emotion regulation in healthy individuals (30f/30m) focusing on the strategy reappraisal. Additionally, potential sex effects have been explored. Narcissism was assessed using self-report measures and emotion regulation with self-report questionnaires as well as an experimental regulation task. During this task, participants were presented with pictures of sad/happy faces with the instruction to indicate their subjective emotions via button press. Depending on the condition, participants either indicated their natural response or applied cognitive control strategies to regulate their own subjective emotions. Results indicate no relationship between grandiose and vulnerable narcissism and emotion regulation ability, irrespective of sex. Individuals high on vulnerable narcissism use the maladaptive regulation strategy suppression more frequently than individuals with low expressions. Individuals high on grandiose narcissism, in contrast, seem to avoid the suppression of positive emotions and do not express negative emotions in an uncontrolled manner. Interestingly, while grandiose narcissism was not associated with depressive symptoms, vulnerable narcissism correlated positively with depressive symptoms and anhedonia. Findings of this study underline the need to differentiate between grandiose and vulnerable manifestations of narcissism. Against our expectation, narcissism was not related to emotion regulation performance. In line with previous research, grandiose narcissism seems less harmful for mental health, while vulnerable narcissism is associated with psychological problems and the use of rather maladaptive emotion regulation strategies, i.e., suppression. Future research should investigate the relationship between pathological narcissism and emotion regulation also by extending the scope to other relevant regulation strategies.

## Introduction

The concept of narcissism has gained increasing attention, for instance, in the context of leadership, and has been linked to positive factors such as high achievement, innovation, and charisma as well as to negative factors such as lack of concern for others, risk to company’s reputation (e.g., fraud), and arrogance (for review see Grijalva et al., 2015; Fatfouta, 2019). Usually, a narcissistic person is described by an inflated self-view, dominance, and exploitive and self-serving behavior. Such definition, however, neglects important aspects of narcissism such as vulnerability, interpersonal hypersensitivity, depressiveness, and social withdrawal (Pincus and Lukowitsky, 2010). Research indeed revealed two manifestations of narcissism, namely grandiosity and vulnerability, which seem to have divergent implications for regulatory styles and mental health (Kealy et al., 2012; Marčinko et al., 2014; Krizan and Herlache, 2018; Kaufman et al., 2020). How these two manifestations relate to the ability to regulate emotions, which is essential for well-being, has only been scarcely investigated.

Krizan and Herlache (2018) suggested a unified conceptual framework, the narcissism spectrum model, describing narcissism in terms of dimensions of individual tendencies that vary in severity and their presentation (grandiosity vs vulnerability). In more detail, the model suggests entitled self-importance as common core of grandiose and vulnerable narcissism with grandiosity and vulnerability reflecting excesses in approach- and avoidance-orientations, respectively. Accordingly, individuals high in grandiose narcissism tend to seek and satisfy self-aggrandizing and rewarding goals. They use self-regulatory styles focusing on self-enhancement rather than on costs, which is manifested in assertive, arrogant and exhibitionist social behavior (Krizan and Herlache, 2018). In line, research revealed a link between grandiosity and high extraversion, dominance, overconfidence, and positive affect (Rhodewalt et al., 1998; Cain et al., 2008; Fulford et al., 2008; Miller et al., 2011; Krizan and Herlache, 2018; Kaufman et al., 2020). Individuals high in vulnerable narcissism, in contrast, tend to detect and combat threats to the self-image (i.e., fight-flight responses). They use self-regulatory styles which excessively focus on self-protection revealed through dismissive, shy, but ultimately volatile social behavior (Krizan and Herlache, 2018). Vulnerable narcissism is further related to low self-esteem and feelings of self-worth, anxieties, neuroticism, and depressiveness (Marčinko et al., 2014; Krizan and Herlache, 2018; Kaufman et al., 2020). The inhibited temperament of individuals high in vulnerable narcissism often leads to a frustration of narcissistic needs for admiration and success (Krizan and Herlache, 2018). Vulnerable narcissism has been even linked to homicidal ideation, parasuicidal behavior, and suicide attempts (for review see Pincus and Lukowitsky, 2010) which underlines the importance to differentiate between grandiose and vulnerable themes in research and also clinical care.

The relationship between narcissism and mental health has been widely discussed. In order to stay psychologically healthy, adequate emotion regulation is crucial (Gross, 1998; Eftekhari et al., 2009; Aldao et al., 2010; Werner and Gross, 2010). The most frequently investigated emotion regulation strategy is reappraisal, an essential component of cognitive behavioral therapy (Beck et al., 1979). Reappraisal refers to the ability to change how a person thinks about a situation in order to alter the emotional response (Gross, 1998; Gross and Thompson, 2007). It is considered a very effective emotion regulation strategy (Webb et al., 2012), as it intervenes early in the process of emotion generation (Gross, 1998; Gross and Thompson, 2007). Research indeed demonstrated numerous positive effects of reappraisal such as increased positive and decreased negative emotions (Troy et al., 2018; Webb et al., 2012) and better psychological health (e.g., Kraaij et al., 2002). Furthermore, experimental studies revealed that individuals high in habitual reappraisal show less physiological reactivity in response to anger induction (Mauss et al., 2007). When it comes to narcissism, little is known about reappraisal or emotion regulation in general. Distinguishing between grandiose and vulnerable narcissism, the latter in particular appears to be associated with regulatory difficulties (e.g., Zhang et al., 2015). Zhang et al. (2015) examined the association between overt and covert narcissism (often used interchangeable with “grandiose” and “vulnerable” narcissism, Pincus and Lukowitsky, 2010) and emotion regulation difficulties. The authors additionally examined respiratory sinus arrhythmia as index of an individual’s physiological regulation and related it to difficulties in habitual emotion regulation. The study revealed that covert/vulnerable narcissism was related to overall emotion regulation difficulties, non-acceptance of emotional responses, impulse control difficulties, limited access to emotion regulation strategies, and a lack of emotional clarity, while individuals high in overt/grandiose narcissism had more emotional awareness and clarity. Respiratory sinus arrhythmia reactivity in response to stress induction moderated the association between covert/vulnerable narcissism and emotion regulation difficulties. In line, Given-Wilson and colleagues revealed that vulnerable but not grandiose narcissism is related to affect dysregulation (Given-Wilson et al., 2011). Further research showed altered physiological arousal in response to stress in individuals scoring high on narcissism (Kelsey et al., 2001) and differentiated psychophysiological reactivity during coping between overt/grandiose and covert/vulnerable narcissists (Kelsey et al., 2002). Previous research investigated predominantly habitual emotion regulation by means of self-report questionnaires. However, the frequency of how often a person applies specific emotion regulation strategies does not imply how successful a person can regulate emotions. For this reason, it is important to include measures of the actual emotion regulation ability in order to interpret emotion regulation difficulties. The current study therefore aims to examine the association between grandiose and vulnerable narcissism and emotion regulation, particularly focusing on the emotion regulation strategy reappraisal. We differentiate between the use of reappraisal in everyday life (i.e., habitual reappraisal) and the ability to regulate emotions by means of reappraisal when instructed to do so (i.e., reappraisal ability). Furthermore, we aim to assess the relationship between grandiose and vulnerable narcissism and depressive symptoms. Based on findings of Zhang et al. (2015) and Given-Wilson et al. (2011), we expect that vulnerable narcissism is linked to emotion regulation difficulties as reflected in decreased use of reappraisal in daily life, a reduced emotion regulation ability, and increased depressive symptoms. Grandiose narcissism, in turn, is expected to be not related to emotion regulation difficulties and depressive symptoms.

## Materials and Methods

### Participants

The sample comprised 60 healthy participants (30 females, 30 males; see Table 1 for more details) with no previous/current mental disorder assessed with the structured clinical interview according to the DSM-IV (Wittchen et al., 1997). Participants were recruited via flyers and announcements in online portals (e.g., University’s blackboard) and were Caucasians since the emotion regulation task included only Caucasian stimuli.

**TABLE 1:** Sociodemographic and clinical characteristics, emotion regulation, and narcissism for the total sample as well as for females and males separately [presented as mean; *n* = 60 (30 females, 30 males)].

	Mean females (SD)	Mean males (SD)	*p*	Mean total (SD)	Range total (min–max)
Age (in years)	34.30 (10.31)	35.10 (10.08)	0.778	34.70 (10.12)	22–54
Education (in years)	14.17 (2.47)	14.80 (3.04)	0.389	14.48 (2.77)	10–21
Verbal Intelligence (WST)	32.87 (3.28)	34.37 (2.68)	0.056	110.08 (10.08)	92–139
Depression (BDI-II)	3.23 (2.86)	3.13 (3.36)	0.560	3.18 (3.10)	0−11(0−63)
Anhedonia (MASQ)	44.97 (11.42)	48.77 (12.59)	0.145	46.87 (12.07)	24−78(22−110)
Reappraisal (ERQ)	28.17 (5.68)	41.87 (9.95)	0.911	27.95 (5.98)	12−37(6−42)
Suppression (ERQ)	12.07 (4.43)	14.13 (5.35)	0.159	13.10 (4.98)	4−24(4−28)
Uncontrolled expression NEG (ERI)	7.40 (3.71)	5.87 (4.07)	0.070	6.63 (3.94)	0−15(0−20)
Controlled expression NEG (ERI)	14.70 (3.67)	11.57 (3.84)	0.003**	13.13 (4.04)	5−20(0−20)
Empathic suppression NEG (ERI)	7.67 (2.68)	8.13 (3.57)	0.817	7.90 (3.14)	0−16(0−16)
Distraction NEG (ERI)	10.72 (1.74)	9.70 (2.51)	0.653	9.88 (2.15)	4−14(0−16)
Reappraisal NEG (ERI)	9.73 (3.08)	9.50 (2.98)	0.655	9.62 (3.01)	3−15(0−16)
Uncontrolled expression POS (ERI)	10.60 (2.27)	8.50 (3.05)	0.006**	9.55 (2.87)	1−14(0−16)
Controlled expression POS (ERI)	12.23 (2.73)	10.33 (2.71)	0.018*	11.28 (2.86)	5−16(0−16)
Empathic suppression POS (ERI)	5.77 (2.81)	5.77 (3.42)	0.929	5.77 (3.11)	0−16(0−16)
Distraction POS (ERI)	1.90 (1.71)	2.07 (2.86)	0.494	1.98 (2.34)	0−12(0−16)
**Grandiose Narcissism**					
NPI-15 Total	7.77 (1.19)	8.10 (1.77)	0.665	7.93 (1.51)	5−12(0−15)
NPI-15 Leadership/Authority	3.37 (1.27)	4.33 (1.45)	0.012*	3.85 (1.44)	1−7(0−9)
NPI-15 Grandiose Exhibitionism	2.60 (0.72)	2.10 (0.92)	0.020*	2.35 (0.86)	0−3(0−3)
NPI-15 Entitlement/Exploitiveness	0.73 (0.45)	0.53 (0.51)	0.111	0.63 (0.49)	0−1(0−1)
**Vulnerable Narcissism**					
NI-R Total	112.27 (24.14)	117.93 (19.68)	0.264	115.10 (22.02)	63−162(42−210)
NI-R Admiration	39.50 (10.87)	43.70 (11.72)	0.203	41.60 (11.40)	19−80(17−85)
NI-R Pretension	27.27 (6.05)	28.50 (4.46)	0.419	27.88 (5.30)	13−38(18−90)
NI-R Mistrust	37.97 (11.27)	40.67 (9.60)	0.254	39.32 (11.47)	20−59(15−75)

BDI-II, Beck Depression Inventory II; ERI, Emotion Regulation Inventory; ERQ, Emotion Regulation Questionnaire; IQ, intelligence quotient; MASQ, Mood and Anxiety Symptom Scale; NEG, Negative emotions; NI-R, Narcissism Inventory Revised; NPI-15, Narcisstic Personality Inventory, 15 items; POS, positive emotions; SD, standard deviation; WST, Wortschatztest. *P*-values indicate sex differences, *p ≤ 0.05, **p ≤ 0.01.

All participants gave written informed consent and received financial compensation (10 Euro). The study was approved by the local ethics committee of the Medical Faculty of the RWTH Aachen University and conducted according to the Declaration of Helsinki.

### Questionnaires

Participants completed measures assessing verbal intelligence (Wortschatztest, WST; Schmidt and Metzler, 1992), depressive symptoms (Beck Depression Inventory II, BDI-II; Hautzinger et al., 2006) and anhedonia (Mood and Anxiety Symptom Scale, MASQ; Watson and Clark, 1991). In order to investigate emotion regulation strategies applied in daily life (i.e., habitual emotion regulation), the emotion regulation questionnaire (ERQ; Gross and John, 2003; Abler and Kessler, 2009) and the emotion regulation inventory (ERI; König, 2011) were used.

To quantify grandiose narcissism, participants completed the 15 item version of the narcissistic personality inventory (NPI-15; Raskin and Hall, 1979; Raskin and Terry, 1988; Schütz et al., 2004). Items have a forced-choice format each consisting of a narcissistic and a non-narcissistic option. The total score ranges from 0 to 15 with higher scores indicating increased grandiose narcissism. The NPI-15 has frequently been used in research and is sufficiently consistent and stable over time (Schütz et al., 2004; Bertl et al., 2017; Ozimek et al., 2018). In our study, participants reached a mean total score of 7.93 (SD = 1.77) which is relatively high compared to other studies examining students and the general population (Schütz et al., 2004; Ozimek et al., 2018). Ackerman et al. (2011) revealed a three factor structure of the NPI consisting of the subscales Leadership/Authority, Grandiose Exhibitionism, and Entitlement/Exploitiveness. Applying structural equation modeling analysis to several narcissism measures, including the NPI, Ackerman et al. (2011) further suggested that the NPI subscales Leadership/Authority (e.g., “I like to have authority over others”) and Grandiose Exhibitionism (e.g., “I prefer to be the center of attention”) are linked to grandiose narcissism whereas the scale Entitlement/Exploitiveness (e.g., “I find it easy to manipulate people”) represents the key “ingredient” of narcissism (common to grandiose and vulnerable narcissism), reflecting a broader tendency toward antagonism (Ackerman et al., 2011; Krizan and Herlache, 2018). For this reason, we considered the subscales Leadership/Authority and Grandiose Exhibitionism as measures of grandiose narcissism. To provide a full picture of narcissism, we additionally report results for the NPI-15 total score and for the NPI-15 Entitlement/Exploitiveness scale in Tables 1–5.

Vulnerable narcissism has been assessed using a shortened and revised version of the original narcissism inventory (NI-R; Deneke and Hilgenstock, 1998; Neumann and Bierhoff, 2004). The NI-R comprises 42 items examining the classic narcissistic self and idealistic self. Items are rated on a 5-point Likert scale ranging from 1 = “not at all true” to 5 = “completely true.” The total score ranges from 42 to 210 with higher scores indicating increased vulnerable narcissism. Participants in our study reached an average total score of 115.10 and SD = 22.02 (Mean item score = 2.72, SD = 0.51), which is in line with previous studies (Ozimek et al., 2018). The NI-R shows a good internal consistency and validity (Neumann and Bierhoff, 2004; Ozimek et al., 2018). It has been used in several previous studies (e.g., Neumann and Bierhoff, 2004; Ozimek et al., 2018; Rohmann et al., 2012) and has been recommended as a valid measure of vulnerable narcissim (Bierhoff et al., 2019). Recently, Altmann (2017) revealed a three-factor structure of a brief (17-item) version of the NI-R consisting of the subscales Admiration (e.g., “I think others envy my good looks”), Pretension (“I set high moral standards for myself – many others are less strict with themselves”), and Mistrust (“Never show your weakness to others, because they will only take advantage of it”). In accordance with the author’s recommendation, we consider the subscales Pretension and Mistrust as measures of vulnerable narcissism. To provide a full picture of narcissism, we additionally report results for the NI-R total scale and for NI-R Admiration in Tables 1–5.

Table 2 shows correlations between grandiose and vulnerable narcissism as assessed with the NPI-15 and NI-R, respectively.

**TABLE 2:** Spearman correlation coefficients of the association between grandiose and vulnerable narcissism.

		Grandiose narcissism (NPI-15)			
		Total	Leadership/authority	Grandiose exhibitionism	Entitlement/exploitiveness
Vulnerable narcissism (NI-R)	Total	–0.016	–0.008	–0.086	–0.092
	Admiration	–0.022	0.063	–0.234	–0.099
	Pretension	0.010	–0.043	0.052	0.104
	Mistrust	–0.152	–0.193	0.111	–0.177

There are no significant correlations. *p ≤ 0.05.

### Experimental Emotion Regulation Task – Emotion Regulation Ability

In contrast to emotion regulation questionnaires (i.e., ERQ and ERI), which capture self-reported use of specific emotion regulation strategies in everyday life, the actual emotion regulation ability can be measured by means of an experimental task. For this reason, participants performed an emotion regulation task which was successfully implemented in a previous study (Loeffler et al., 2018; Loeffler et al., 2019). Emotion regulation difficulties occur in particular in social interactions. Since facial emotions convey important information in social communication, they offer an ideal possibility to examine social emotion regulation. Therefore, 45 sad and 45 happy Caucasian faces of the FACES database (Ebner et al., 2010) were presented for 4 s on a computer screen. Subsequently, participants indicated via button press how sad (regarding sad faces) or happy (regarding happy faces) they felt on a scale ranging from 1 (not at all) to 8 (very). Faces of the same emotions were grouped into mini-blocks of five trials. The inter-stimulus interval amounted to 2–4 s.

The task consisted of three counterbalanced conditions, implemented in three separate blocks (each condition containing 15 sad and 15 happy faces). In the *view* condition, no regulation was applied and participants should imagine that they encounter the person depicted on the picture on the street or somewhere else. In the two experimental conditions *up-regulation* and *down-regulation* they should imagine that the person on the picture was a close person in order to increase the personal relevance. In the *up-regulation* condition, participants were additionally instructed to imagine that the person on the picture was sad/happy because of them whereas in the *down-regulation* condition they should imagine they had nothing to do with the emotional state of the person on the picture. Stimuli were presented by Presentation Software (Neurobehavioral Systems, Albany, CA, United States) and viewed on a laptop screen.

### Statistical Analysis

#### Habitual Emotion Regulation

The association between grandiose and vulnerable narcissism and the use of cognitive emotion regulation strategies in everyday life was examined by correlating scores (total score and subscale scores) of the NPI-15 (grandiose narcissism) and NI-R (vulnerable narcissism) with reappraisal scores (ERQ and ERI). Due to violations of normal distribution, Spearman correlations have been used. Moreover, we conducted uncorrected exploratory correlations between narcissism scores and strategies additionally assessed with the ERQ and ERI (e.g., suppression) using Spearman correlations. To test for sex differences in habitual emotion regulation, Mann-Whitney *U* tests have been conducted (see Table 1 for details).

#### Emotion Regulation Ability

First, to investigate emotion regulation ability irrespective or narcissism, emotion ratings of the experimental task were averaged and analyzed with a repeated-measures ANOVA with condition (view, up-regulation, down-regulation) and emotion (sad and happy) as within-subject factors and sex (male and female) as between-subjects factor to account for potential sex effects. Next, analyses were repeated with total scores of the NPI-15 and NI-R as covariates. In a final step, analyses were conducted with subscale scores (instead of total scores) of the NPI-15 and NI-R as covariates. Significant effects were followed-up with Bonferroni-corrected pairwise comparisons or with Spearman correlations.

#### Depression/Anhedonia

Furthermore, to describe the relationship between narcissism (NPI-15 and NI-R), depressive symptoms (BDI-II and MASQ), and sex, Spearman correlations were calculated due to violations of normality.

## Results

Findings revealed a stronger expression of grandiose narcissism (but not vulnerable narcissism) in men compared to women (NPI-15 subscale Leadership/Authority and Grandiose Exhibitionism; see Table 1). The following sections describe the association between narcissism and habitual emotion regulation, emotion regulation ability, and depression symptoms.

### Habitual Emotion Regulation

#### Grandiose Narcissism (NPI-15 Leadership/Authority, Grandiose Exhibitionism)

Grandiose narcissism did not significantly correlate with the emotion regulation strategy reappraisal (*p* ≥ 0.089) Exploratory analyses revealed a significant negative correlation between grandiose narcissism (NPI-15 Leadership/Authority) and the empathic suppression of positive emotions (ERI; *r* = −0.277, *p* = 0.032) as well as with the uncontrolled expression of negative emotions (ERI; *r* = −0.317, *p* = 0.014). No further significant correlations emerged (see Table 3 for further details).

**TABLE 3:** Spearman correlation coefficients of the association between narcissism and habitual emotion regulation.

	Grandiose narcissism (NPI-15)				Vulnerable narcissism (NI-R)			
	Total	Leadership/Authority	Grandiose exhibitionism	Entitlement/Exploitiveness	Total	Admiration	Pretension	Mistrust
Reappraisal (ERQ)	–0.138	–0.025	–0.221	0.008	0.055	0.069	0.124	–0.053
Reappraisal NEG (ERI)	–0.032	0.037	0.010	–0.169	0.005	0.106	0.082	–0.053
Suppression (ERQ)	–0.010	–0.100	0.155	0.008	0.331**	0.331**	0.104	0.387**
Uncontrolled expression NEG (ERI)	–0.211	−0.317*	0.073	0.037	0.205	0.146	0.039	0.143
Controlled expression NEG (ERI)	–0.173	–0.198	0.099	0.136	–0.193	–0.187	0.066	–0.245
Empathic suppression NEG (ERI)	0.039	–0.023	0.159	0.020	0.147	–0.102	0.150	0.227
Distraction NEG (ERI)	0.271*	0.223	0.092	0.049	0.039	–0.037	0.163	0.014
Uncontrolled expression POS (ERI)	–0.061	–0.070	–0.139	0.014	0.167	0.183	0.255*	0.005
Controlled expression POS (ERI)	–0.163	–0.031	–0.169	–0.069	0.095	0.058	0.232	–0.012
Empathic suppression POS (ERI)	–0.213	−0.277*	0.150	–0.016	0.148	0.109	0.042	0.227
Distraction POS (ERI)	–0.046	–0.135	0.091	0.078	0.167	0.183	–0.044	0.091

NEG, negative emotions; POS, positive emotions. Significant correlations indicated with *p ≤ 0.05, **p ≤ 0.01

#### Vulnerable Narcissism (NI-R Pretension, Mistrust)

**FIGURE 1:**
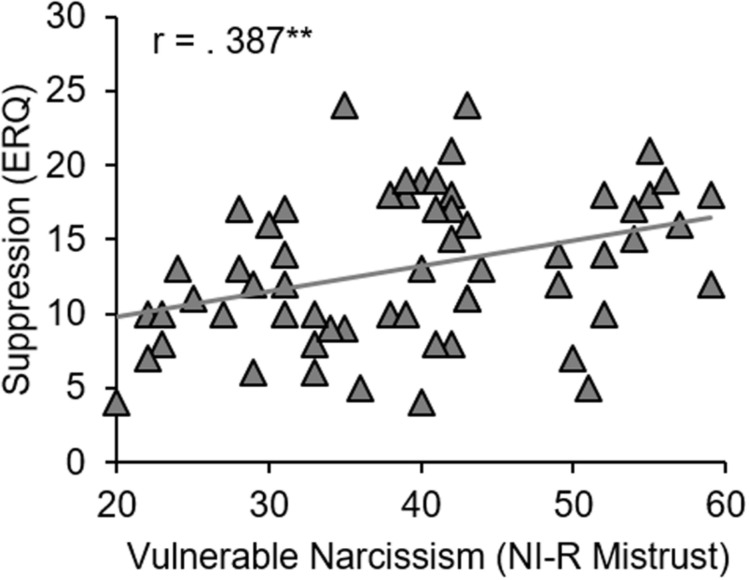
Spearman correlation between the emotion regulation strategy suppression (ERQ) and vulnerable narcissism (NI-R-Mistrust). **p ≤ 0.01.

Similar to grandiose narcissism, vulnerable narcissism was not significantly related to reappraisal (*p* ≥ 0.347). Exploratory analyses, however, revealed a significant positive association between vulnerable narcissism (NI-R Mistrust) and suppression (ERQ; *r* = 0.387, *p* = 0.002; see Figure 1). Moreover, the NI-R subscale Pretension correlated positively with the uncontrolled expression of positive emotions (ERI; *r* = 0.255, *p* = 0.050). No further significant correlations emerged (see Table 3 for further details).

#### Sex

Females applied the emotion regulation strategies “controlled expression of negative emotions” (ERI; *p* = 0.003), “uncontrolled expression of positive emotions” (ERI; *p* = 0.006), and “controlled expression of positive emotions” (ERI; *p* = 0.018) more often than males. No further significant sex differences emerged (all *p* ≥ 0.070, see Table 1 for details).

### Emotion Regulation Ability

#### Emotion Regulation Ability

**FIGURE 2:**
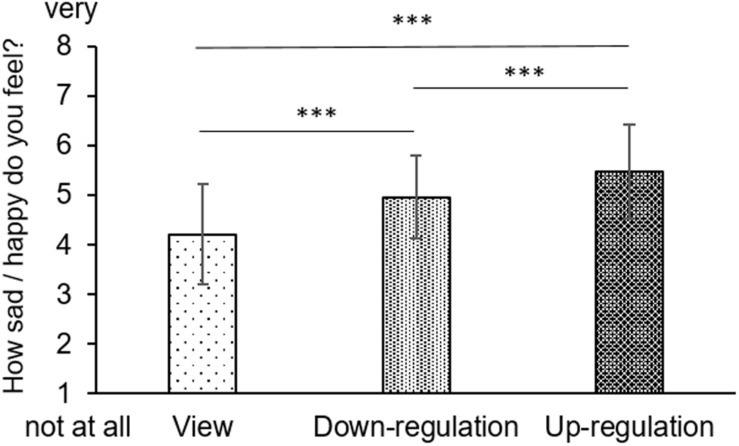
Emotion ratings during the emotion regulation task (mean ratings with standard deviations). ***p ≤ 0.001.

Bonferroni-corrected follow-up pairwise comparisons of a significant main effect of condition [*F*(1.625, 94.267) = 65.238, *p* < 0.001] revealed significant differences between all three conditions (view vs up-regulation: *p* ≤ 0.001, view vs down-regulation: *p* ≤ 0.001, up-regulation vs down-regulation: *p* ≤ 0.001, see Figure 2), confirming successful emotion regulation. Furthermore, there was a significant main effect of emotion [*F*(1, 58) = 42.072, *p* < 0.001] with significantly higher happiness than sadness ratings. This difference between happiness and sadness ratings was particularly pronounced in the view condition as suggested by follow-up pairwise comparisons of a significant condition-by-emotion interaction [*F*(1.868, 108.341) = 8.925, *p* < 0.001]. There were no further significant main effects or interactions (see Table 4 for details).

**TABLE 4:** The association between narcissism and emotion regulation ability.

Emotion regulation ability		
Condition	*F*(1.625, 94.267) = 65.238	*p* < 0.001***
Emotion	*F*(1, 58) = 42.072	*p* < 0.001***
Sex	*F*(1, 58) = 0.235	*p* = 0.630
Condition × Emotion	*F*(1.868, 108.341) = 8.925	*p* < 0.001***
Condition × Sex	*F*(1.625, 94.267) = 0.163	*p* = 0.850
Emotion × Sex	*F*(1, 58) = 0.359	*p* = 0.552
Condition × Emotion × Sex	*F*(1.868, 108.341) = 1.310	*p* = 0.273
**Grandiose narcissism (NPI-15)**		
NPI-15 Total	*F*(1, 56) = 0.144	*p* = 0.706
NPI-15 Total × Condition	*F*(1.626, 91.036) = 0.129	*p* = 0.837
NPI-15 Total × Emotion	*F*(1, 56) = 0.127	*p* = 0.722
NPI-15 Total × Sex	*F*(1, 56) = 0.021	*p* = 0.884
NPI-15 Total × Condition × Emotion	*F*(1.891, 105.874) = 0.216	*p* = 0.794
NPI-15 Total × Condition × Sex	*F*(1.626, 91.036) = 0.069	*p* = 0.900
NPI-15 Total × Emotion × Sex	*F*(1, 56) = 0.330	*p* = 0.568
NPI-15 Total × Condition × Emotion × Sex	*F*(1.891, 105.874) = 1.954	*p* = 0.149
NPI-15 Leadership/Authority	*F*(1, 52) = 0.753	*p* = 0.390
NPI-15 Leadership/Authority × Condition	*F*(1.612, 83.810) = 0.752	*p* = 0.448
NPI-15 Leadership/Authority × Emotion	*F*(1, 52) = 0.001	*p* = 0.999
NPI-15 Leadership/Authority × Sex	*F*(1, 52) = 0.403	*p* = 0.528
NPI-15 Leadership/Authority × Condition × Emotion	*F*(1.892, 98.377) = 0.017	*p* = 0.980
NPI-15 Leadership/Authority × Condition × Sex	*F*(1.612, 83.810) = 0.038	*p* = 0.936
NPI-15 Leadership/Authority × Emotion × Sex	*F*(1, 52) = 0.056	*p* = 0.814
NPI-15 Leadership/Authority × Condition × Emotion × Sex	*F*(1.892, 98.377) = .685	*p* = 0.499
NPI-15 Grandiose Exhibitionism	*F*(1, 52) = 0.028	*p* = 0.868
NPI-15 Grandiose Exhibitionism × Condition	*F*(1.612, 83.810) = 0.750	*p* = 0.449
NPI-15 Grandiose Exhibitionism × Emotion	*F*(1, 52) = 0.342	*p* = 0.561
NPI-15 Grandiose Exhibitionism × Sex	*F*(1, 52) = 0.524	*p* = 0.472
NPI-15 Grandiose Exhibitionism × Condition × Emotion	*F*(1.892, 98.377) = 0.290	*p* = 0.737
NPI-15 Grandiose Exhibitionism × Condition × Sex	*F*(1.612, 83.810) = 2.481	*p* = 0.101
NPI-15 Grandiose Exhibitionism × Emotion × Sex	*F*(1, 52) = 1.746	*p* = 0.192
NPI-15 Grandiose Exhibitionism × Condition × Emotion × Sex	*F*(1.892, 98.377) = 0.047	*p* = 0.948
NPI-15 Entitlement/Exploitiveness	*F*(1, 52) = 0.233	*p* = 0.631
NPI-15 Entitlement/Exploitiveness × Condition	*F*(1.612, 83.810) = 2.724	*p* = 0.083
NPI-15 Entitlement/Exploitiveness × Emotion	*F*(1, 52) = 0.190	*p* = 0.665
NPI-15 Entitlement/Exploitiveness × Sex	*F*(1, 52) = 1.453	*p* = 0.234
NPI-15 Entitlement/Exploitiveness × Condition × Emotion	*F*(1.892, 98.377) = 1.729	*p* = 0.185
NPI-15 Entitlement/Exploitiveness × Condition × Sex	*F*(1.612, 83.810) = 1.495	*p* = 0.231
NPI-15 Entitlement/Exploitiveness × Emotion × Sex	*F*(1, 52) = 1.213	*p* = 0.276
NPI-15 Entitlement/Exploitiveness × Condition × Emotion × Sex	*F*(1.892, 98.377) = 3.398	*p* = 0.037*
**Vulnerable narcissism (NI-R)**		
NI-R Total	*F*(1, 56) = 5.362	*p* = 0.024
NI-R Total × Condition	*F*(1.553, 86.988) = 3.621	*p* = 0.042
NI-R Total × Emotion	*F*(1, 56) = 0.680	*p* = 0.413
NI-R Total × Sex	*F*(1, 56) = 0.519	*p* = 0.474
NI-R Total × Condition × Emotion	*F*(1.862, 104.289) = 0.095	*p* = 0.897
NI-R Total × Condition × Sex	*F*(1.553, 86.988) = 0.437	*p* = 0.597
NI-R Total × Emotion × Sex	*F*(1, 56) = 2.020	*p* = 0.161
NI-R Total × Condition × Emotion × Sex	*F*(1.862, 104.289) = 1.575	*p* = 0.212
NI-R Admiration	*F*(1, 52) = 2.623	*p* = 0.111
NI-R Admiration × Condition	*F*(1.548, 80.505) = 0.162	*p* = 0.795
NI-R Admiration × Emotion	*F*(1, 52) = 0.210	*p* = 0.649
NI-R Admiration × Sex	*F*(1, 52) = 1.390	*p* = 0.244
NI-R Admiration × Condition × Emotion	*F*(1.876, 97.531) = 0.452	*p* = 0.625
NI-R Admiration × Condition × Sex	*F*(1.548, 80.505) = 0.398	*p* = 0.620
NI-R Admiration × Emotion × Sex	*F*(1, 52) = 0.069	*p* = 0.794
NI-R Admiration × Condition × Emotion × Sex	*F*(1.876, 97.531) = 0.919	*p* = 0.397
NI-R Pretension	*F*(1, 52) = 3.523	*p* = 0.066
NI-R Pretension × Condition	*F*(1.548, 80.505) = 0.243	*p* = 0.727
NI-R Pretension × Emotion	*F*(1, 52) = 0.643	*p* = 0.426
NI-R Pretension × Sex	*F*(1, 52) = 2.050	*p* = 0.158
NI-R Pretension × Condition × Emotion	*F*(1.876, 97.531) = 0.791	*p* = 0.449
NI-R Pretension × Condition × Sex	*F*(1.548, 80.505) = 0.259	*p* = 0.715
NI-R Pretension × Emotion × Sex	*F*(1, 52) = 9.115	*p* = 0.004**
NI-R Pretension × Condition × Emotion × Sex	*F*(1.876, 97.531) = 0.181	*p* = 0.821
NI-R Mistrust	*F*(1, 52) = 0.350	*p* = 0.556
NI-R Mistrust × Condition	*F*(1.548, 80.505) = 2.086	*p* = 0.142
NI-R Mistrust × Emotion	*F*(1, 52) = 0.087	*p* = 0.769
NI-R Mistrust × Sex	*F*(1, 52) = 0.235	*p* = 0.630
NI-R Mistrust × Condition × Emotion	*F*(1.876, 97.531) = 0.004	*p* = 0.995
NI-R Mistrust × Condition × Sex	*F*(1.548, 80.505) = 1.034	*p* = 0.344
NI-R Mistrust × Emotion × Sex	*F*(1, 52) = 0.001	*p* = 0.972
NI-R Mistrust × Condition × Emotion × Sex	*F*(1.876, 97.531) = 0.429	*p* = 0.640

Findings indicate main effects and interactions of the repeated measures AN(C)OVAS. Follow-up Spearman correlations of the significant fourway-interaction between NPI-15 Entitlement/Exploitiveness, Condition, Emotion, and Sex revealed that only within females (not males), ratings in the view condition correlated negatively with narcissism scores. In more detail, females with higher Entitlement/Exploitiveness scores indicated lower subjective emotion ratings when instructed to indicate their natural response to sad and happy faces (i.e., they seem to be less emotionally affected by the emotional state of others). *p ≤ 0.05, **p ≤ 0.01, and ***p ≤ 0.001.

#### Grandiose Narcissism (NPI-15 Leadership/Authority, Grandiose Exhibitionism)

Repeated-measures ANCOVA revealed no significant main effect or interactions of grandiose narcissism (see Table 4 for further details).

#### Vulnerable Narcissism (NI-R Pretension, Mistrust)

Repeated-measures ANCOVA showed a significant three-way interaction between NI-R Pretension, emotion, and sex [*F*(1, 52) = 9.115, *p* = 0.004]. Follow-up Spearman correlations unveiled that only within females [*r* = 0.607, *p* < 0.001], but not males (*r* = −0.150, *p* = 0.430), narcissism scores correlated positively with happiness ratings (Fisher’s *z* = 3.14, *p* = 0.001). No further main effects or interactions of vulnerable narcissism were significant (see Table 4 for further details).

### Depression/Anhedonia

#### Grandiose Narcissism (NPI-15 Leadership/Authority, Grandiose Exhibitionism

There was no significant association between grandiose narcissism and depression (BDI-II) or anhedonia (MASQ) (see Table 5 for details).

**TABLE 5:** Spearman correlation coefficients of the association between narcissism and depression / anhedonia symptoms.

	BDI-II	MASQ-Anhedonia
**Grandiose Narcissism**		
NPI-15 Total	0.062	0.020
NPI-15 Leadership/Authority	0.052	–0.150
NPI-15 Grandiose Exhibitionism	0.003	–0.162
NPI-15 Entitlement/Exploitiveness	–0.110	0.048
**Vulnerable Narcissism**		
NI-R Total	0.354**	0.248
NI-R Admiration	0.212	0.184
NI-R Pretension	0.038	–0.066
NI-R Mistrust	0.357*	0.319*

Significant correlations indicated with *p ≤ 0.05, **p ≤ 0.01.

#### Vulnerable Narcissism (NI-R Pretension, Mistrust)

**FIGURE 3:**
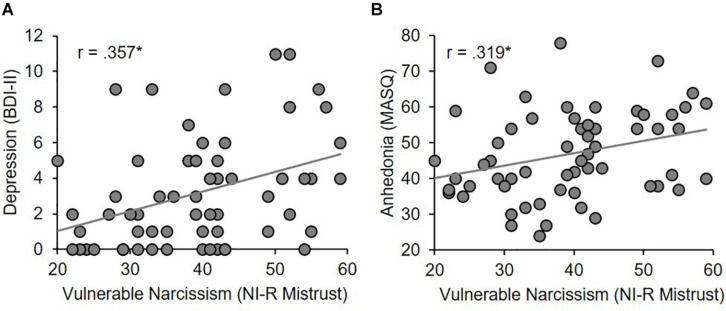
Spearman correlations between depressive symptoms (**A**, BDI-II) and anhedonia (**B**, MASQ) and vulnerable narcissism (NI-R Mistrust). *p ≤ 0.05.

Vulnerable narcissism (NI-R Mistrust) was positively related to depressive symptoms (BDI-II; *r* = 0.357, *p* = 0.005) and anhedonia (MASQ; *r* = 0.319, *p* = 0.013) (Figure 3; see Table 5 for details).

#### Sex Effects

There were no significant sex differences in depressive symptoms (BDI-II; *p* = 0.560) or anhedonia (MASQ; *p* = 0.226; see Table 1 for details).

## Discussion

The current study investigated the association between two forms of narcissism, namely grandiose and vulnerable narcissism, and emotion regulation in a sample of healthy individuals. We differentiated between habitual reappraisal, i.e., how often a person self-reports to use reappraisal in daily life, and reappraisal ability, i.e., the ability to regulate emotions using reappraisal when instructed to do so. Results revealed no significant association between (grandiose and vulnerable) narcissism and emotion regulation ability as well as the habitual use of reappraisal. However, exploratory analyses showed that vulnerable narcissism was related to a greater use of the emotion regulation strategy suppression whereas individuals high on grandiose narcissism seem to refrain from using this strategy. Furthermore, only vulnerable narcissism was linked to depressive symptoms.

Against our expectation, there was no significant association between vulnerable narcissism and habitual reappraisal. On the one hand, this could be due to the fact that the sample consisted of healthy participants without any history of mental disorders. Perhaps the (reduced) use of specific emotion regulation strategies (i.e., reappraisal) only becomes apparent in (sub)clinical samples. Future research should therefore investigate emotion regulation in pathological narcissism. On the other hand, even healthy individuals with high narcissistic expressions may show a (dis)favor for specific emotion regulation strategies, though probably not regarding the regulation strategy investigated here. Supporting this assumption, the current study revealed that healthy individuals with higher scores in vulnerable narcissism use suppression more frequently in daily life than individuals with low scores. This strategy refers to the suppression of an emotional reaction (e.g., facial expressions) once a full emotion has already been elicited (Gross and Thompson, 2007). Due to its limited effects on subjective emotions and unwanted “side-effects” such as increased cardiovascular arousal (Gross and Levenson, 1997), suppression is often considered as rather maladaptive. In this sense, our findings support the notion that vulnerable narcissism appears to be associated with less adaptive emotion regulation. The finding that specifically the NI-R subscale Mistrust relates to a greater use of emotion suppression is in line with previous research (Altmann, 2017) and appears plausible as Mistrust is characterized by the expectation to be exploited by others (Altmann, 2017), which might require the deception of own emotions. Important to note, NI-R Mistrust does not cover the full range of vulnerable traits. Future studies should therefore extent findings to other aspects of vulnerable narcissism such as neuroticism, contingency and withdrawal. In line with our expectation of grandiose narcissism being related to less emotion regulation disturbances, our findings imply that individuals high in grandiose narcissism seem to avoid the suppression of positive emotions. Since grandiose narcissism is linked to an approach-orientation toward rewards it is not surprising that individuals with high expressions of grandiosity do not suppress positive feelings. The link between reduced use of this rather maladaptive strategy and grandiose narcissism was, however, only significant for the NPI-15 subscale Leadership/Authority, which has been claimed to reflect rather adaptive aspects of grandiosity (Ackerman et al., 2011). It comprises self-perceived leadership ability, social potency, and dominance and could be linked to the fearless dominance aspect of psychopathy as well as to self-esteem (Ackerman et al., 2011). It is important to mention that the mere use of certain (mal)adaptive strategies (e.g., suppression) does not necessarily indicate (dys)functional emotion regulation. In certain challenging contexts, suppression of the emotional response may be even desirable. Difficulties arise when emotion regulation strategies are inflexibly applied. Future research should therefore examine how individuals react to different situations to determine whether the strategies are applied flexibly and appropriate to the context. The emotional state of narcissists has been shown to be determined by their approach versus avoidance behavior (Czarna et al., 2018), which goes along with positive or negative emotionality, respectively (Elliot and Thrash, 2002). This is in line with the narcissism spectrum model (Krizan and Herlache, 2018) stating that vulnerable narcissists are rather avoidance-oriented and sensitive to threats, while grandiose narcissists are approach-oriented and sensitive to rewards. In support of this model, our findings related vulnerable narcissism to the avoidance-oriented emotion regulation strategy suppression whereas grandiose narcissism was linked to a reduced use of it.

In line with previous findings (Given-Wilson et al., 2011; Zhang et al., 2015), our results further revealed that vulnerable narcissism, but not grandiose narcissism, is associated with depressive symptoms. In more detail, higher expressions in NI-R Mistrust (vulnerable narcissism) were related to higher self-reported depressive symptoms and anhedonia. Mistrust refers to “competitive rivalry, devaluing others if they are not a source of admiration, and concealing one’s needs and faults” and could be linked to a reduced life satisfaction (Altmann, 2017). It might therefore reflect maladaptive aspects of vulnerable narcissism. The NI-R Pretension, which relates to high moral standards and a desire to be admired for it (Altmann, 2017), was unrelated to depressive symptoms suggesting rather adaptive aspects of vulnerability. This interpretation is in line with previous findings of a positive, though small association with life satisfaction (Altmann, 2017). Importantly, only healthy participants with low depressive symptoms which have no clinical relevance have been included in this study. For this reason, future studies should examine the relationship between depression and narcissism in mild to moderately depressed individuals. In agreement with our results, previous research supports, however, an association between vulnerable narcissism and depression (Miller et al., 2011) as well as with characteristics predisposing to mental problems such as low self-esteem (Boldero et al., 2015). Furthermore, it has been shown that individuals at risk for depression tend to apply suppression rather than reappraisal (Ehring et al., 2010) suggesting an association between maladaptive emotion regulation use, which seems to be characteristic for vulnerable narcissism and mental health problems.

Important to note, we cannot necessarily deduce from our findings on habitual emotion regulation whether a person has emotion regulation difficulties, but only how often a certain (mal)adaptive regulation strategy is applied. We have therefore additionally examined the actual ability to regulate emotions by means of an experimental task. Similarly to habitual reappraisal, grandiose narcissism was not related to reappraisal ability, which is in line with findings of Zhang et al. (2015). Surprisingly, neither was vulnerable narcissism significantly associated with reappraisal ability. A particular strength of our study was the assessment of both negative and positive emotion regulation but both without significant relations to narcissism, making valence-specific regulation deficits in narcissism unlikely. However, females with high expressions of vulnerable narcissism generally indicated higher happiness ratings during the emotion regulation task. Specifically, women high in NI-R Pretension reported high subjective happiness, which is in line with our suggestion that Pretension might reflect rather adaptive aspects of vulnerability. As mentioned earlier, our findings of a lacking association between narcissism and emotion regulation ability may be due to the inclusion of only healthy participants. Furthermore, non-significant results might be also the result of a relatively small sample and potentially lack of statistical power. Although it limits the number of participants included, our experimental assessment of regulation abilities is an important strength of our study, complementing previous studies on self-reported regulation. Likewise, the investigated regulation strategy, namely reappraisal, may account for the results. Since the “cognitive wave” in psychotherapy, there has been a strong focus on cognitive processes in emotion regulation and their significance for mental health. Nevertheless, other regulation strategies need to be considered as well. It has been suggested, for instance, that pathological narcissism might be specifically linked to externalizing regulation strategies such as substance use (Pincus and Lukowitsky, 2010).

The current study makes an important contribution to a better understanding of emotion regulation processes in vulnerable and grandiose narcissism. Our findings underline the need to examine both phenotypes since vulnerable narcissism (specifically Mistrust) seems to be related to rather maladaptive emotion regulation strategies and mental health problems while no such associations emerged for grandiose narcissism. However, it has been questioned whether these subtypes can really be separated or whether they are merely extremes of one narcissistic dimension between which narcissists can oscillate depending on environmental changes (e.g., experiences of insult or success; Lammers et al., 2013; Lammers and Doering, 2018). In line, Ronningstam (2009) highlights an oscillation between grandiose and vulnerable states and further proposes that narcissistic personality disorder is characterized by “a pervasive pattern of fluctuating and vulnerable self-esteem ranging from grandiosity and assertiveness to inferiority or insecurity, with self-enhancing and self-serving interpersonal behavior, and intense reactions to perceived threats” (p. 118). But even if vulnerable and grandiose narcissism represent two extremes of a narcissism dimension, it is mandatory to consider both phenotypes, both in research and health care. Otherwise, there is a risk of an underrepresentation of vulnerable narcissism, which may lead to a biased diagnosis of narcissism and in the worst case non-optimal treatment of individuals with predominantly vulnerable narcissistic traits.

## Data Availability Statement

The dataset for this study will not be made publicly available because we do not have an ethics votum for sharing the data.

## Ethics Statement

The study involves human participants and was reviewed and approved by the local ethics committee of the Medical Faculty of the RWTH Aachen University. The participants provided their written informed consent to participate in this study.

## Author Contributions

All authors contributed to the conception of the study. AH collected the data. LL and AH analyzed the data and drafted the manuscript. All authors contributed to the interpretation of the results. Furthermore, all authors critically revised the manuscript and approved the publication of its content.

## Conflict of Interest

The authors declare that the research was conducted in the absence of any commercial or financial relationships that could be construed as a potential conflict of interest.
